# Regulation of autophagy by coordinated action of mTORC1 and protein phosphatase 2A

**DOI:** 10.1038/ncomms9048

**Published:** 2015-08-27

**Authors:** Pui-Mun Wong, Yan Feng, Junru Wang, Rong Shi, Xuejun Jiang

**Affiliations:** 1 Cell Biology Program, Memorial Sloan-Kettering Cancer Center, 1275 York Avenue, New York, New York 10065, USA; 2 Xiangya School of Medicine, Central South University, Hunan 410008, China

## Abstract

Autophagy is a cellular catabolic process critical for cell viability and homoeostasis. Inhibition of mammalian target of rapamycin (mTOR) complex-1 (mTORC1) activates autophagy. A puzzling observation is that amino acid starvation triggers more rapid autophagy than pharmacological inhibition of mTORC1, although they both block mTORC1 activity with similar kinetics. Here we find that in addition to mTORC1 inactivation, starvation also causes an increase in phosphatase activity towards ULK1, an mTORC1 substrate whose dephosphorylation is required for autophagy induction. We identify the starvation-stimulated phosphatase for ULK1 as the PP2A–B55α complex. Treatment of cells with starvation but not mTORC1 inhibitors triggers dissociation of PP2A from its inhibitor Alpha4. Furthermore, pancreatic ductal adenocarcinoma cells, whose growth depends on high basal autophagy, possess stronger basal phosphatase activity towards ULK1 and require ULK1 for sustained anchorage-independent growth. Taken together, concurrent mTORC1 inactivation and PP2A–B55α stimulation fuel ULK1-dependent autophagy.

## Abstract (web summary)

Starvation rapidly triggers autophagy by inactivating mTORC1 signalling, however mTORC1 inhibition does not fully recapitulate this rapid induction. Wong *et al.* identify a parallel starvation-senstive pathway regulating autophagy, mediated by protein phosphatase 2A-dependent dephosphorylation of ULK1.

## Graphical abstract

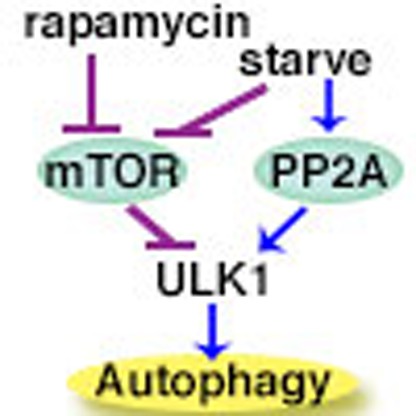

Autophagy is a highly conserved catabolic pathway that targets selective proteins and organelles within the cell for lysosomal degradation. During autophagy, an isolation membrane extends to encapsulate cargo for degradation. The growing isolation membrane seals to form a double membrane vesicle termed an autophagosome, which delivers its contents to lysosomes^1^. As a crucial homoeostatic mechanism, autophagy is involved in multiple physiological processes, and its deregulation has been implicated in many diseases^2^,^3^,^4^,^5^. While basal levels of autophagy are generally present in cells, autophagy can be strongly activated in response to a variety of cell stresses, such as endoplasmic reticulum stress, hypoxia and nutrient starvation. The autophagy pathway is mediated by ATG (autophagy-related) proteins that make up several distinct complexes, among which the ULK1 complex and the VPS34 complex function as important gatekeepers for the induction of autophagy^1^,^6^,^7^,^8^.

The ULK1 complex comprises of regulatory subunits ATG13, FIP200, ATG101 and the core Ser/Thr kinase ULK1. ULK1 is essential for autophagy induced by amino acid starvation (referred to simply as starvation herein) and is directly regulated by energy and nutrient-sensing kinases mTORC1 and AMP-activated protein kinase (AMPK)^9^,^10^,^11^,^12^,^13^. The reversible phosphorylation of ULK1 is a central signalling mechanism through which starvation-induced autophagy is regulated. On sensing a decrease in amino acid levels, the activity of mTORC1 is suppressed and ULK1 is concurrently activated^9^,^10^. ULK1 is a direct substrate of mTORC1 at multiple sites including S637 and S757 in murine ULK1, and undergoes global dephosphorylation upon starvation or pharmacological inhibition of mTOR^9^,^12^,^14^. Interestingly, S637 can also be phosphorylated by AMPK^12^. While the kinases regulating ULK1 phosphorylation are well documented, relatively little is known about the phosphatases involved in this process.

For the 428 putative serine/threonine (Ser/Thr) kinases in the human genome, only ∼30 Ser/Thr phosphatases are known^15^,^16^. While phosphatases were once believed to be passive and promiscuous enzymes, this view is slowly being overturned with the continual discovery of interacting proteins that bind to phosphatases to regulate their activity^17^,^18^. In this study we monitor two mTOR sites on ULK1 and find that more than one phosphatase acts in opposition to mTOR to dephosphorylate ULK1 during starvation. We identify one of the phosphatases to be the PP2A–B55α complex and demonstrate that the phosphatase is activated upon starvation. Under fed conditions the PP2A catalytic subunit is sequestered by inhibitory protein Alpha4, keeping it in an inactive state. Starvation triggers the release of PP2A from this latent complex, resulting in rapid dephosphorylation of ULK1 and autophagy induction. Furthermore, we find that this phosphatase activity is abnormally high in pancreatic ductal adenocarcinoma cells that require high basal autophagy for viability. We propose that activation of the phosphatase activity towards ULK1 represents a mechanism that allows cancer cells to activate a strong autophagy flux without turning off mTOR activity, thus achieving optimal growth and survival capability.

## Results

### Starvation activates a phosphatase activity towards ULK1

**Figure 1:**
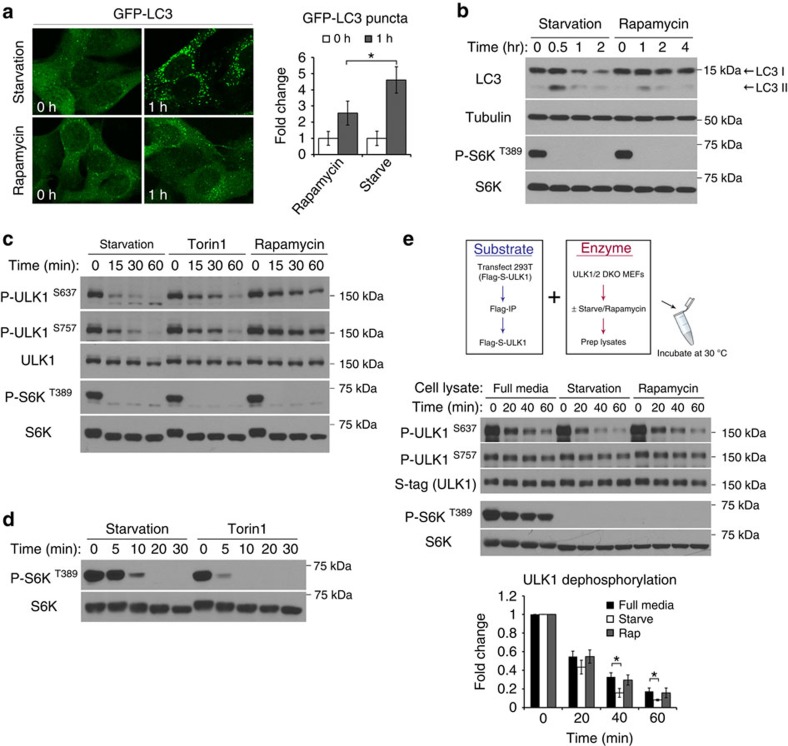
Amino acid starvation stimulates a protein phosphatase for ULK1. (**a**,**b**) Starvation induces a stronger autophagic response than rapamycin. In **a**, MEFs stably expressing GFP-LC3 were incubated in starvation media or treated with 1-μM rapamycin for the indicated time. Representative images from three independent experiments shown, average number of puncta per cell was quantitated and expressed as fold change relative to 0 h (fold change ±s.d., *n*=30. two-tail Student’s *t*-test, *P<0.05). In **b**, MEFs were lysed at the indicated time points and immunoblotted for endogenous LC3 and other proteins as indicated. (**c**) Kinetics of mTOR substrate dephosphorylation in response to starvation or pharmacological inhibition of mTOR. MEFs were incubated in starvation media or media containing 1 μM of either Torin1 or rapamycin, and lysed at the indicated time points. Lysates were analysed for the endogenous levels and phosphorylation states of the specified proteins. (**d**) Torin1 shuts down mTOR activity more efficiently than starvation. MEFs were incubated in starvation media or media containing 1 μM Torin1 for the indicated amount of time. (**e**) Starvation increases phosphatase activity for ULK1 S637 in MEFs. Upper panel shows a schematic representation of the *in vitro* phosphatase assay. Middle panel shows outcome of the phosphatase assay comparing lysates from starved, fed or rapamycin-treated cells. Reactions were terminated at the indicated time and analysed by western blotting for ULK1 phosphorylation status. S-tag and total S6K are loading controls for the amount of substrate and enzyme, respectively, in each reaction. Lower panel shows quantitation of ULK1 S637 phosphorylation relative to time 0 from four independent experiments (fold change±s.d., *n*=4. two-tail Student’s *t*-test, *P<0.05).

Suppression of mTORC1 activity induces ULK1 complex-dependent autophagy^9^,^10^. Given the central role of mTORC1 in regulating starvation-induced autophagy, it was puzzling to observe that starvation could induce a faster autophagic response compared with pharmacological inhibition of mTORC1. The autophagy marker LC3 was tagged with green fluorescent protein (GFP) for fluorescence microscopy. As shown in Fig. 1a,b, compared with rapamycin treatment, starvation induced more GFP-LC3 puncta in mouse embryonic fibroblasts (MEFs), it also induced more rapid conversion of LC3 I to LC3 II (thus more rapid decrease of LC3 I). These results suggest that in addition to suppressing mTORC1, nutrient starvation may also engage other mechanisms for autophagy activation.

To gain more insights into this process, we compared the kinetics of ULK1 dephosphorylation triggered by starvation versus pharmacological inhibition of mTOR. We monitored ULK1 phosphorylation status using two phospho-specific antibodies recognizing S637 and S757 phosphorylation (Supplementary Fig. 1a,b). The kinetics of starvation-induced ULK1 dephosphorylation was more rapid compared with that induced by rapamycin (Fig. 1c), which can explain why starvation induces stronger autophagy than rapamycin. Mechanistically, as rapamycin is an indirect/allosteric inhibitor of mTORC1, we considered the possibility that the difference in dephosphorylation rate may be due to incomplete inhibition of mTORC1 by rapamycin^19^,^20^. In such a scenario, high-affinity mTORC1 substrates such as 4EBP1 would remain phosphorylated in the presence of rapamycin while weaker substrates such as S6K would be dephosphorylated^21^. However, Torin1, a potent ATP-competitive inhibitor of mTOR, also led to slower ULK1 dephosphorylation than starvation (Fig. 1c), even though Torin1 treatment was more effective at shutting down mTORC1 activity than starvation as detected at earlier time points (Fig. 1d).

As mTORC1 inactivation is insufficient to account for the rapid dephosphorylation and thereby activation of ULK1 during starvation, we hypothesized that starvation activates a phosphatase towards ULK1. Indeed, in a dose-dependent manner, whole cell lysate from starved MEFs had higher phosphatase activity towards ULK1 than that from un-starved MEFs or rapamycin-treated MEFs (Fig. 1e and Supplementary Fig. 1c). Importantly, this starvation-regulated phosphatase activity towards ULK1 was also observed in other cell types, including fibrosarcoma HT1080 cells (Supplementary Fig. 1d).

### Phosphatase activity is required for autophagy induction

**Figure 2:**
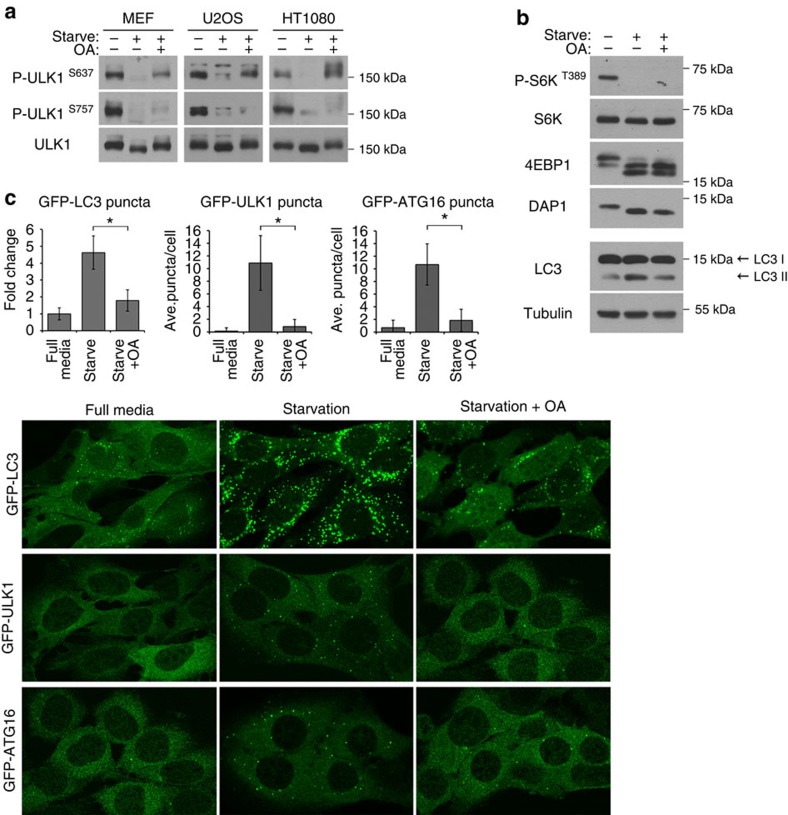
Okadaic acid inhibits autophagy. (**a**) Okadaic acid (OA) inhibits the dephosphorylation of ULK1 at S637 but not S757. MEF, U2OS or HT1080 cells were incubated in starvation media or starvation media with 200 nM OA for 1 h. Phosphorylation of S637 and S757 on ULK1 was monitored using site specific phospho-antibodies. (**b**) MEFs were treated as in **a** and lysates were probed for three reported mTOR substrates (S6K, 4EBP1 and DAP1) and endogenous LC3. (**c**) OA inhibits starvation-induced autophagy. MEFs stably expressing GFP-LC3, GFP-ULK1 or GFP-ATG16 were incubated in starvation media or starvation media containing 200 nM OA for 1 h. Cells were fixed and imaged for GFP-LC3, GFP-ULK1 or GFP-ATG16 translocation. Representative images from two independent experiments shown. Average number of puncta per cell were quantitated and expressed as such or as fold change relative to full media (fold change or mean±s.d., *n*=20. two-tail Student’s *t*-test, *P<0.05).

To identify the starvation-stimulated phosphatase for ULK1, we first tested the effect of Okadaic acid (OA), a pharmacological inhibitor of the phosphoprotein phosphatase family^22^. In a dose-dependent manner, OA treatment blocked both starvation-induced dephosphorylation of ULK1 at S637 and that of AKT, which is known to be sensitive to OA^23^, but it had no effect on dephosphorylation of other mTORC1 substrates such as S6K, 4EBP1 and DAP1 (Fig. 2a,b and Supplementary Fig. 2). Remarkably, dephosphorylation at a second site on ULK1 (S757) was not blocked by OA treatment either, suggesting that more than one phosphatase is involved in the regulation of ULK1. This observation was consistent in all tested cell lines, ruling out the possibility of a cell line specific artifact (Fig. 2a). Correlating with the specific inhibition of S637 dephosphorylation of ULK1, OA treatment greatly diminished starvation-induced translocation of the ULK1 complex and the downstream ATG16 complex to punctate structures (Fig. 2c). As expected, OA-induced block of these two early events in autophagosome formation translated to a significant block in autophagy response to starvation (Fig. 2b,c).

### PP2A dephosphorylates ULK1 at S637

**Figure 3:**
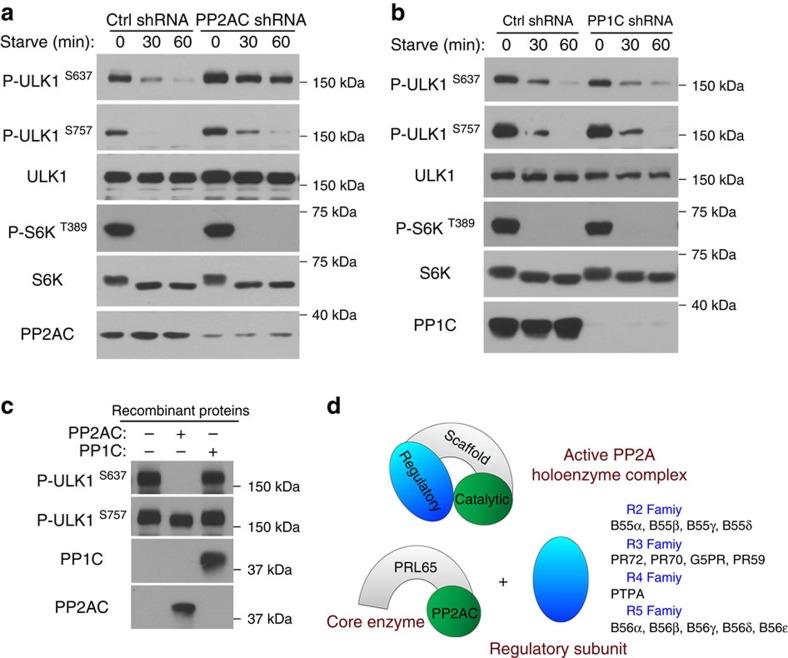
PP2A but not PP1 dephosphorylates ULK1 at S637. (**a**) PP2A is required for dephosphorylation of ULK1 at S637. MEFs were transduced with lentivirus containing control shRNA or shRNA targeting the catalytic subunit of PP2A (PP2AC). 48–72 h post transduction, cells were incubated in starvation media for the indicated time and lysed for immunoblotting. (**b**) PP1C is not required for ULK1 dephosphorylation. MEF cells were treated as in **a** with shRNA targeting the catalytic subunit of PP1 (PP1C). (**c**) PP2AC directly dephosphorylates ULK1 S637 *in vitro*. Catalytic subunit of PP1 (PP1C) was included as a control. Flag-S–ULK1 substrate was incubated with 50 nM of recombinant PP2AC or PP1C at 30 °C for 20 min. (**d**) Diagram representing the PP2A Ser/Thr phosphatase complex. Regulatory subunits confer substrate specificity to the core enzyme.

OA can inhibit multiple protein phosphatases from the phosphoprotein phosphatase family, including PP1, PP2A, PP4, PP5 and PP6 (ref. 22). Among these OA-sensitive phosphatases, PP1 and PP2A are most abundant and ubiquitously expressed in cells^22^, hence we started our search by targeting these two phosphatases. Knockdown of either the catalytic subunit (PP2AC) or scaffolding subunit (PRL65) of PP2A inhibited the dephosphorylation of ULK1 at S637 (Fig. 3a and Supplementary Fig. 3) whereas knockdown of PP1 catalytic subunit (PP1C) had no effect on this event despite a near complete depletion of PP1C (Fig. 3b). Likewise, recombinant PP2AC but not PP1C could dephosphorylate ULK1 at S637 (Fig. 3c). Consistent with ULK1 behaviour in OA-treated cells (Fig. 2a), knockdown of PP2A had no effect on S757 dephosphorylation (Fig. 3a and Supplementary Fig. 3). Furthermore, recombinant PP2AC could not dephosphorylate ULK1 at S757 either (Fig. 3c). These results further confirm that differential phospho-sites of ULK1, although all phosphorylated by the same kinase mTORC1, are regulated by distinct protein phosphatases.

### Identification of PP2A regulatory subunit

**Figure 4:**
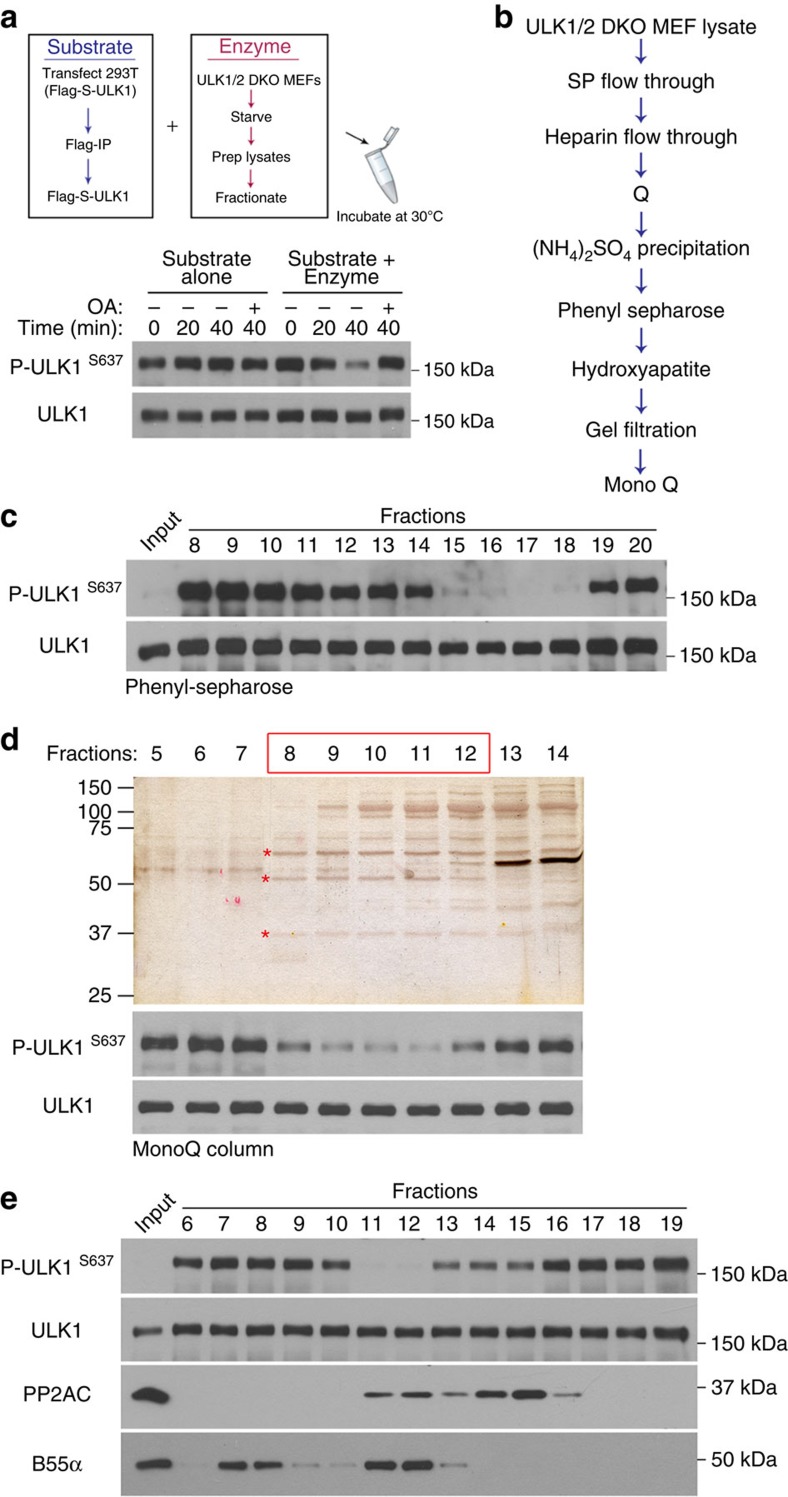
Purification of PP2A regulatory subunit. (**a**) Set up of *in vitro* phosphatase assay. The substrate (Flag-S–ULK1) is stable in the reaction over time and dephosphorylated only in the presence of an enzyme source (total or fractionated cell extract). (**b**) Purification scheme for the identification of the PP2A regulatory subunit from starved ULK1/2 DKO cell lysate. (**c**) Example of phosphatase assay reaction during PP2A regulatory subunit purification. Fractions from Phenyl Sepharose column were assessed for activity against ULK1. Active fractions (15–18) were combined as input for the next purification step. (**d**) Silver staining (top) and phosphatase activity assay (bottom) of fractions from the final purification step. Active fractions are boxed in red. Indicated bands were excised for mass spectrometric analysis. (**e**) Western blot showing distribution of PP2AC, B55α and ULK1 S637 phosphatase activity after a Q-column.

PP2A is a multi-functional protein subjected to tight regulation in the cell. As an active complex, it is a heterotrimer consisting of the catalytic subunit (PP2AC), the scaffolding subunit (PRL65), and a variable regulatory subunit that confers substrate specificity and thus dictates the biological function of PP2A (Fig. 3d)^17^,^24^,^25^. To identify the regulatory subunit targeting ULK1, we performed biochemical fractionation in a series of seven chromatographic steps (Fig. 4b) using the extracts of starved ULK1/2-double knockout (DKO) MEFs as the starting material. Fractions from each column were analysed for phosphatase activity against ULK1 as in Fig. 4a,c. From relatively early steps in the purification, it was clear that only a subpopulation of PP2A was responsible for ULK1 dephosphorylation (Supplementary Fig. 4a). Fractions from the final column were analysed by SDS–polyacrylamide gel electrophoresis (PAGE) followed by silver staining (Fig. 4d). Three distinct bands at 36, 65 and 55 kDa correlated with the phosphatase activity for S637 site of ULK1. As expected, mass spectrometry identified the 36- and 65-kDa bands as the PP2A catalytic and scaffolding subunit, respectively (Supplementary Fig. 5a,b). The 55-kDa band was the PP2A regulatory subunit B55α (Supplementary Fig. 5c). Consistently, western blot analysis demonstrated that during fractionation, B55α correlated perfectly with the PP2AC-containing fractions that were active against ULK1 (Fig. 4e, fractions 11 and 12), while adjoining fractions containing other forms of PP2AC such as that containing regulatory subunit B′γ (Fig. 4e, fractions 14 and 15; and Supplementary Fig. 4b,c) had little activity against ULK1. Being a regulatory subunit with no catalytic activity on its own, fractions containing B55α but no PP2AC had no activity against ULK1 (Fig. 4e, fractions 7 and 8).

### B55α directs PP2A phosphatase activity against ULK1

**Figure 5:**
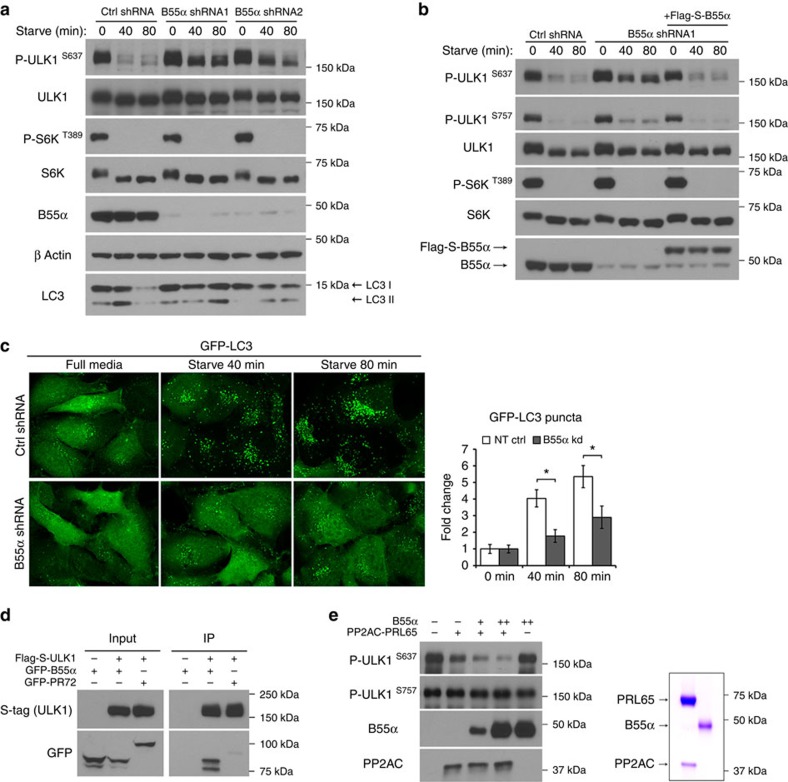
Regulatory subunit B55α directs PP2A phosphatase activity against ULK1. (**a**) B55α is required for ULK1 S637 dephosphorylation. HT1080 cells stably expressing tet-inducible control shRNA, shRNA targeting the 3′UTR region of B55α (shRNA1) or shRNA targeting the coding region of B55α (shRNA2) were generated. Cells were cultured in 100 ng ml^−1^ Doxycycline and incubated in starvation media for the indicated amount of time. (**b**) HT1080 cells harbouring control shRNA, B55α shRNA1 or B55α shRNA1 and stable over- expression of Flag-S–B55α were incubated in starvation media for the indicated time and analysed for ULK1 dephosphorylation. (**c**) Knockdown of B55α blocks starvation-induced autophagy as monitored by florescence imaging of GFP-LC3 in HT1080 cells. Representative images from two independent experiments shown, average number of puncta per cell were quantitated and expressed as fold change relative to 0 min (fold change±s.d., *n*=20. two-tail Student’s *t*-test, *P<0.05). (**d**) ULK1 interacts preferentially with B55α. 293Ts were co-transfected with Flag-S–ULK1 and GFP-B55α or GFP-PR72 (regulatory subunit from a different family). Cells were lysed and incubated with S-beads to pull down ULK1. (**e**) B55α stimulates PP2A activity towards ULK1. *In vitro* phosphatase assay was carried out using 5 nM of recombinant PP2AC co-purified with PRL65 and increasing amounts of B55α (10 nM or 40 nM). Coomassie brilliant blue staining of recombinant protein preparations is shown on the right.

To confirm the requirement of B55α in cells, we knockeddown B55α using two independent shRNA sequences. Both efficiently blocked dephosphorylation at S637 and resulted in a significant block in starvation-induced autophagy, as assessed by turnover of endogenous LC3 as well as GFP-LC3 puncta formation (Fig. 5a,c). Re-expression of B55α (shRNA-resistant and Flag-S-tagged) could reverse the effects of B55α knockdown on ULK1 S637 dephosphorylation, indicating that the block in dephosphorylation is specifically due to reduction in B55α levels (Fig. 5b). Crystal structure data of the PP2A heterotrimer suggests that the regulatory subunit facilitates substrate recruitment^26^. In line with this, ULK1 preferentially interacted with B55α but not an unrelated regulatory subunit from another family (PR72) as assessed by a co-immunoprecipitation experiment (Fig. 5d).

To demonstrate that B55α makes PP2A a more efficient phosphatase towards ULK1, we reconstituted the phosphatase reaction *in vitro* using recombinant proteins of PP2AC, PRL65 and B55α. Recombinant PP2AC and PRL65 were co-expressed in insect cells using baculoviral vectors while B55α was expressed and purified to homogeneity separately. On its own, PP2AC is sufficient to dephosphorylate ULK1 *in vitro* when used at high concentrations (Fig. 3c). However, when used at a low concentration, we could see a dose-dependent stimulatory effect of B55α on PP2A activity towards ULK1 (Fig. 5e). B55α alone when used at its highest dose had no phosphatase activity against ULK1. All these results indicate that B55α is the PP2A regulatory subunit for dephosphorylating ULK1 at S637.

### A latent population of PP2A is activated during starvation

How does starvation stimulate the activity of PP2A towards ULK1? PP2AC expression is tightly regulated in the cell^27^, and short-term starvation does not cause change in the expression of PP2A complex.

**Figure 6:**
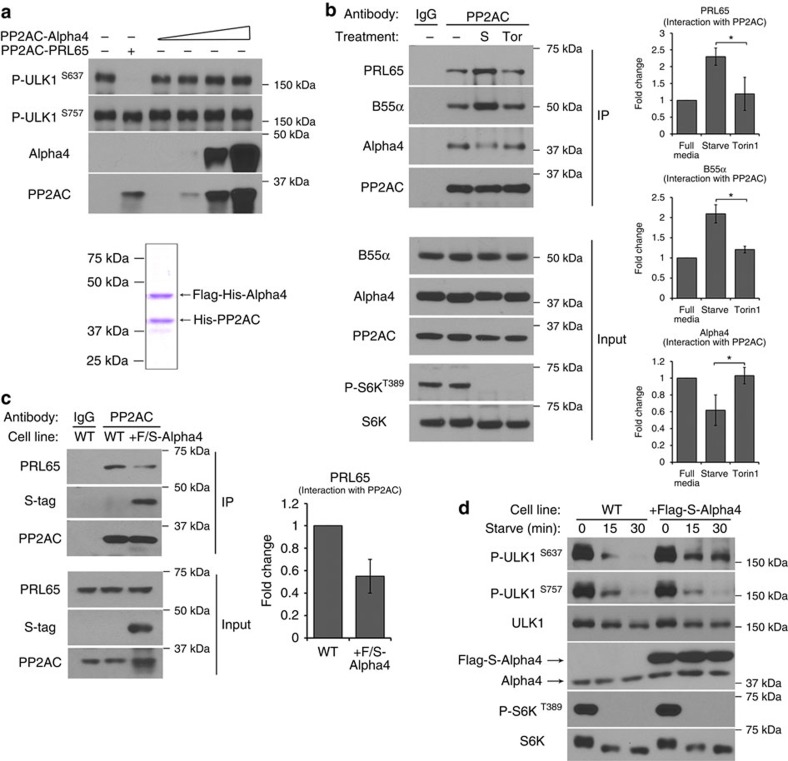
Starvation stimulates release of PP2A catalytic subunit from inhibitory protein Alpha4. (**a**) Recombinant PP2AC–Alpha4 complex is inactive. Phosphatase assay was carried out *in vitro* using increasing amounts of recombinant PP2AC (3–90 nM) co-purified with Alpha4. Recombinant PP2AC-PRL65 complex (20 nM) was used as a control. Coomassie brilliant blue staining of recombinant PP2AC–Alpha4 is shown below. (**b**) Starvation induces dissociation of PP2AC from Alpha4. 293T cells were starved or incubated in complete media containing 1 μM Torin1 for 2 h. Cell lysate was prepared, crosslinked with DSP and incubated with the indicated antibodies to pull down endogenous PP2A complexes for immunoblot analysis. Quantitation of co-IPed proteins relative to full media is shown on the right. (fold change±s.d., *n*=3. two-tail Student’s *t*-test, *P<0.05) (**c**) Alpha4 overexpression reduces PRL65 binding to PP2AC. 293T cells were transfected with Flag-S-Alpha4. Cells were lysed, and incubated with antibodies to pull down endogenous PP2AC. Immunoblotting was carried out to monitor the amount of PRL65 interacting with PP2AC. Panel on the right shows quantitation of PRL65 relative to untransfected cells. (fold change±s.d., *n*=3) (**d**) Alpha4 overexpression blocks ULK1 S637 dephosphorylation. MEF cells stably overexpressing Flag-S-Alpha4 were incubated in complete or starvation media for the indicated amount of time.

In yeast, Pph21 (PP2AC homologue) is regulated by Tap42 in a nutrient-dependent manner^28^. Tap42 is a highly conserved regulator of PP2AC with a mammalian homologue, Alpha4, that has been implicated in mediating cell survival during glutamine starvation^29^,^30^. Recent studies indicate that PP2AC is inactive when bound to Alpha4 (refs 29, 31). Indeed, in an *in vitro* phosphatase assay, the recombinant PP2A–Alpha4 complex could not dephosphorylate ULK1 (Fig. 6a). To further confirm that the Alpha4-containing PP2A complex is inactive in cells, we used a potent small molecule phosphatase inhibitor, microcystine-LR (MCLR) conjugated to agarose beads to isolate endogenous PP2AC complexes from MEFs. MCLR binds to the catalytic site of PP2AC when the enzyme is in an active conformation^32^,^33^. Interestingly, the MCLR beads pulled down PP2A holo-enzyme complex containing the scaffolding (PRL65) and regulatory subunits but did not pull down PP2A complex containing Alpha4 (Supplementary Fig. 6a), indicating that Alpha4-associated PP2AC is inactive in cells. This is in agreement with partial crystal structure data of PP2AC showing that the conformation of the catalytic site is greatly altered when PP2AC is bound to Alpha4 (ref. 31). As a control, immunoprecipitation of PP2AC could pull down Alpha4 (Supplementary Fig. 6a).

In yeast, the Pph21–Tap42 (PP2AC–Alpha4 homologues) interaction is sensitive to rapamycin^28^.

We performed co-immuno precipitation to assess if the PP2AC–Alpha4 interaction was altered by starvation or pharmacological inhibition of mTOR. A clear decrease in the interaction of PP2AC with Alpha4 was detected in starved cells but not in cells treated with Torin1 (Fig. 6b and Supplementary Fig. 6b). Notably, we could detect a concurrent increase in association with PRL65 and B55α during starvation when crosslinking reagent dithiobis (succinimidyl propionate) (DSP) was used to stabilize the complex for immunoprecipitation (Fig. 6b). This result indicates that starvation induces release of PP2AC from latent complexes containing Alpha4 to form active complexes containing PRL65 and regulatory subunits including B55α.

To further demonstrate that Alpha4 can affect the dynamics of active PP2A complex formation, we overexpressed Flag-S doubly tagged Alpha4 in 293T cells and assessed the effect on active PP2A complex formation by immune-precipitation of endogenous PP2AC. Compared with control cells, in Alpha4 overexpressing cells, less PRL65 was co-precipitated with PP2AC (Fig. 6c). We also generated a stable MEF cell line overexpressing Alpha4 in order to assess if this has any functional consequence on starvation-induced ULK1 dephosphorylation. Overexpression of Alpha4 reduced the rate of dephosphorylation at ULK1 S637, consistent with its role as a negative regulator of PP2A activity (Fig. 6d). In agreement with our earlier observation, dephosphorylation at S757 was not affected by Alpha4 overexpression. We were unable to do the converse experiment by RNAi as knockdown of Alpha4 results in depletion of PP2AC expression and cell death, as reported previously^29^,^34^.

### ‘Autophagy-addicted’ cells have high phosphatase activity

**Figure 7:**
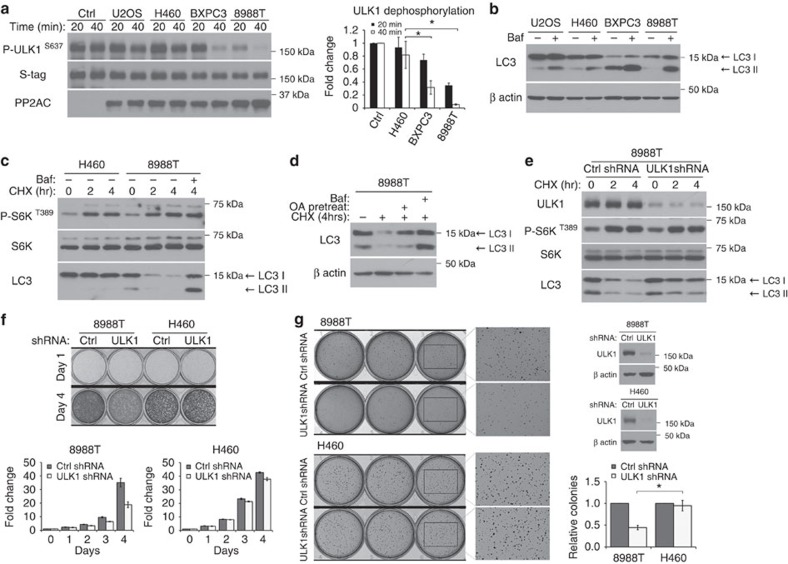
Phosphatase activity drives high basal levels of autophagy in pancreatic ductal adenocarcinoma cell lines to support cancer cell growth. (**a**) Pancreatic ductal adenocarcinoma (PDAC) cell lines have high phosphatase activity. *In vitro* phosphatase assay was carried out using Flag-S–ULK1 as a substrate and 10 μg of total cell lysates from indicated cancer cell lines. Left panel shows immunoblot of ULK1 S637 dephosphorylation, and right panel shows quantitation (fold change ± s.d., *n*=3. two-tail Student’s *t*-test, *P<0.05). (**b**,**c**) PDAC cell lines have high basal autophagy. In **b**, cell lines were kept in complete media in the presence or absence of 20 nM bafilomycin (Baf) for 90 min and lysed for immunoblotting of endogenous LC3. In **c**, PDAC cell line 8988T and control cell line H460 were kept in complete media with 20 μg ml^−1^ cycloheximide (CHX) for the indicated time. Where indicated, 10 nM Baf was added at the start of CHX treatment. (**d**) Phosphatase activity is required for basal turnover of LC3. 8988T was treated as in **c**. To assess phosphatase involvement, cells were pretreated with 200 nM okadaic acid in full media for 2 h and removed at the start of CHX treatment. (**e**) ULK1 complex is required for basal LC3 turnover. 8988T cells were transduced with control shRNA or shRNA targeting ULK1. Cells were treated with 20 μg ml^−1^ CHX for the indicated time and immunoblotted for endogenous LC3. (**f**) ULK1 complex is required for robust proliferation of 8988T cells. Equal number of 8988T or H460 cells stably expressing control shRNA or shRNA targeting ULK1 were seeded on day 0. Upper panel shows cells fixed and stained with crystal violet on day 1 and day 4 respectively. Histograms on the lower panel show quantitation of crystal violet stain over time, relative to day 0. Error bars are standard deviation from triplicates. (**g**) ULK1 complex is required for sustained anchorage-independent growth of 8988T. Cell lines in **f** were used in a soft agar assay. Representative images and knockdown efficiencies are shown. Histogram shows quantitation of colonies in each cell line relative to control shRNA (relative colony number±s.d., *n*=3. two-tail Student’s *t*-test, *P<0.05).

Stimulating autophagy via an mTORC1-independent mechanism could be highly relevant to cancer, considering that some cancers are ‘addicted’ to autophagy, namely, they require strong basal autophagy for viability and growth^35^,^36^,^37^. Recent reports indicate that pancreatic cancer cells with mutant Ras fall within this category^35^,^36^. We examined whether basal autophagy in these cancer cells may be driven by upregulated phosphatase activity for ULK1 while maintaining mTORC1 function intact. As shown in Fig. 7a, cell lysates of pancreatic ductal adenocarcinoma (PDAC) cell lines BXPC3 and 8988T had significantly higher phosphatase activity towards ULK1 S637 site compared with control cancer cell lines U2OS (osteosarcoma) and H460 (non-small cell lung cancer) that are not ‘addicted’ to autophagy. Interestingly, we observed higher expression of regulatory subunit B55α in BXPC3 cells and lower levels of Alpha4 in 8988T cells (Supplementary Fig. 7a), correlating with the high phosphatase activity against ULK1 in these cell lines.

We confirmed high basal levels of autophagy in these cell lines using two complimentary assays. In the first assay, we treated cells with bafilomycin to block degradation of LC3 and observed more dramatic accumulation of LC3 form II in the two PDAC cell lines (Fig. 7b and Supplementary Fig. 7b). In the second assay, we blocked synthesis of new LC3 using translational inhibitor cycloheximide and tracked the degradation of existing LC3 over time. A significantly faster rate of LC3 turnover was detected in both PDAC cell lines compared with control cell line H460 (Fig. 7c,d and Supplementary Fig. 7c). The LC3 turnover in both the cell lines could be blocked by the addition of bafilomycin, consistent with degradation occurring through the autophagosome-lysosome pathway. Importantly, mTOR remained active during the course of cycloheximide treatment (Fig. 7c,e and Supplementary Fig. 7c), indicating that basal autophagy in these cell lines was occurring independent of mTOR inactivation.

Subsequently, we confirmed that both PP2A activity and the ULK1 complex are required for high basal autophagy in PDAC cell line 8988T. Treatment with OA or knockdown of ULK1 complex components (either ULK1 or FIP200) blocked basal turnover of LC3 (Fig. 7d,e and Supplementary Fig. 7d). Knockdown of PP2AC also blocked basal turnover of LC3 (Supplementary Fig. 7e). Furthermore, knockdown of ULK1 reduced proliferation and anchorage-independent growth of 8988T cells without affecting that of the H460 cells (Fig. 7f, g). Hence, high basal autophagy can contribute to the fitness of 8988T cells in a PP2A and ULK1 complex-dependent manner.

## Discussion

In summary, this study has revealed an intriguing mechanism that controls the phosphorylation status, and thus the autophagy function of ULK1. Activation of ULK1 requires its dephosphorylation, which usually occurs after suppression of its kinase mTORC1 under various autophagy-inducing conditions. Thus far the focus has been on the role of mTORC1 in ULK1 regulation. Here, we show that the rapid dephosphorylation of ULK1 triggered by amino acid starvation is not simply a passive consequence of mTOR inactivation alone; stimulation of a specific protein phosphatase for ULK1, PP2A–B55α, by inducing its dissociation from the inhibitory protein Alpha4, is also a critical regulatory event.

This novel mechanism has clear implications in cancer biology. It has been proposed that autophagy can support cancer growth by alleviating metabolic stress^38^,^39^. Knockdown of autophagy-related genes has also been shown to impact tumour growth in several cancer models^36^,^40^,^41^. Here, we demonstrate that ‘autophagy-addicted’ PDAC cell line 8988T requires both PP2A activity and the ULK1 complex to maintain high basal levels of autophagy, rapid proliferation and sustained anchorage-independent growth. That ULK1-dependent autophagy can be promoted by stimulating the protein phosphatase targeting ULK1 without suppressing mTORC1 can explain the seemingly contradictory co-existence of intact mTORC1 function and strong autophagy activity in various cancers. This mechanism allows cancer cells to simultaneously reap the benefits of both mTOR signalling and autophagy activation.

This study is also conceptually important for signal transduction events regulated by protein phosphorylation. While much attention has been given to the regulation of protein kinases, we demonstrate that a protein phosphatase can also be actively regulated to modulate signalling output. Furthermore, the mechanism in play is particularly elegant as multiple layers of specificity can be observed in this case. First, for different substrates (for example, ULK1, 4EBP1, DAP1 and S6K) of the same protein kinase (mTORC1), distinct protein phosphatases are responsible for the reverse, dephosphorylation reaction. Second and more strikingly, even for individual phosphorylation sites on the same substrate (for example, S637 and S757 sites of ULK1, both can be phosphorylated by mTORC1), distinct protein phosphatases may be involved as well.

Many questions remain to be answered. For example, the phosphatase for the S757 site of ULK1, which could be another crucial modulator for ULK1-dependent autophagy, is not yet known. In addition, although starvation can induce dissociation of Alpha4 from PP2AC, the underlying mechanism is not clear. Analogous to its kinase counterpart mTOR, which is subjected to multiple layers of regulation^42^,^43^,^44^,^45^,^46^,^47^, PP2A may also sense nutrient and growth signalling through a complex network of positive and negative regulators.

## Methods

### Reagents and antibodies

Rabbit anti-LC3b (L7543; 1:3,000), mouse anti-β actin Clone AC-15 (A1978; 1:5,000), mouse anti-B55α Clone 2G9 (SAB4200241; 1:4,000) were from Sigma. Mouse anti-α tubulin (clone DM1A; 1:4,000) was from CalBiochem. Rabbit anti-phos S6K (Thr389; 9205; 1:3,000), Rabbit anti-S6K (2708; 1:3,000), Rabbit anti-4EBP1 (9452; 1:2,000), rabbit anti-PRL65 Clone 81G5 (2041; 1:1,000), rabbit anti-DAP1 (2282; 1:2,000), rabbit anti-His Tag (2365; 1:1,000), rabbit anti-B subunit (2290; 1:4,000–1:10,000) were from Cell Signaling. Rabbit anti-phos-ULK1 (S637; 1:4,000) and rabbit anti-phos-ULK1 (S757; 1:4,000) were a generous gift from Dr Xiaodong Wang, National Institute of Biological Science, Beijing, China. N.B. corresponding sites in human ULK1 are S638 and S758. Rabbit anti-total ULK1 (A7481; 1:3,000) used to detect mouse ULK1 was purchased from Sigma. Rabbit anti-total ULK1 (sc33182; 1:1,000) used to detect human ULK1, rabbit anti-PP2AC (sc130237; 1:1,000) and mouse anti-PP1C (sc7482; 1:3,000) were from Santa Cruz. Mouse anti-S-tag antibody (71549-3; 1:2,000) was from Novagen. Mouse anti-PP2AC clone 1D6 (05-421; 1:3,000–1:8,000) was from Millipore. Mouse anti-Alpha4 Clone 5F6 (05-930; 1:3,000–1:8,000) was from Upstate cell signalling solutions. Rapamycin was purchased from Enzo Life sciences (A-275). Torin1 was purchased from Tocris Bioscience. OA was purchased from Sigma (O9381) or Enzo Life sciences (ALX-350-003) with each showing identical effects on ULK1 dephosphorylation. Cycloheximide was purchased from Calbiochem (239–763) and Bafilomycin A1 (B1793) was purchased from Sigma.

### Cell culture

MEFs, U2OS, HT1080 and 8988T cell lines were cultured in DMEM, supplemented with 10% fetal bovine serum, 2 mM L-Glutamine and 1 × penicillin-streptomycin at 37 °C, 5% CO_2_. H460 and BXPC3 cell lines were grown in RPMI medium, supplemented with 10% fetal bovine serum, 2 mM L-Glutamine and 1 × penicillin-streptomycin. H460 and 8988T cell lines were a kind gift from Dr Alec Kimmelman, Dana Farber Cancer Institute, Boston, USA. ULK1/2 DKO MEF cells were courtesy of Dr Craig Thompson, Memorial Sloan-Kettering Cancer Center, New York, USA. All other cell lines were from American Type Culture Collection. Stable expression of transgenes such as GFP-LC3, Flag-S-B55α, Flag-S-Alpha4 and Flag-S–PP2AC or shRNA cassettes were carried out by retroviral or lentiviral transduction. Lentiviral or retroviral constructs were co-transfected with packaging vectors into 293Ts for virus production. Retro/lentivirus preparations were passed through a 0.45 μM polyethersulfone membrane filter (PES filter) and supplemented with polybrene before being used to transduce cells.

### Treatment conditions and cell lysis

For amino acid starvation experiments, cells were washed twice and incubated in DMEM lacking amino acids and serum for the indicated amount of time. For OA treatments to inhibit phosphoprotein phosphatase family phosphatases, as OA is a hydrophobic inhibitor, cells were pretreated with 200-nM OA in full media before switching to starvation media with 200-nM OA to allow time for cellular uptake of OA. Rapamycin and Torin1 treatments to inhibit mTOR activity were both done at a final concentration of 1 μM in complete media. To assess basal autophagy, a cycloheximide chase experiment was carried out. Cells were treated with translational inhibitor cycloheximide (used at a final concentration of 20 μg ml^−1^ in complete media) to block synthesis of nascent LC3. Turnover of existing LC3 was tracked over time by SDS–PAGE analysis. Lysosomal inhibitor Bafilomycin A1 was used at a final concentration of 10 or 20 nM as indicated to assess autophagy flux.

All samples prepared solely for western analysis were washed twice in cold PBS(−) and lysed in RIPA buffer (50 mM Tris pH 7.5, 150 mM NaCl, 1% Triton X-100, 0.5% sodium deoxycholate and 0.1% SDS) supplemented with protease inhibitors and phosphatase inhibitors (P0044 and P5726 from Sigma) unless otherwise stated. Protein concentration was determined using Bio-Rad Protein Assay Dye reagent (#500-0006). Most experiments were done at least three times with a minority of experiments done a minimum of two times when reproducibility was clear. Full scans related to respective figures are shown in Supplementary Fig. 8.

### Fluorescence microscopy

Cells stably expressing the GFP-tagged protein for visualization were plated onto glass coverslips 24 h before the experiment. Following treatment, cells were fixed in 3.7% formaldehyde for 20 min. Coverslips were washed and mounted onto microscope slides using ProLong Gold antifade reagent with DAPI (Molecular probes). Slides were visualized on a Nikon Eclipse TE2000-U microscope or a Nikon Eclipse Ti-U confocal Microscope. Acquired images were processed using Adobe Photoshop.

### *In vitro* dephosphorylation assay

Generation of substrate (Flag-S–ULK1) used in phosphatase assays: ULK1 substrate was generated in a mammalian system to ensure proper phosphorylation. pBabe-Flag-S–ULK1 construct generated previously^9^ was transfected into 293T cells using Lipofectamine2000 (Invitrogen) according to the manufacturer’s recommendations. Cells were split 24 h post transfection to prevent overgrowth and harvested 48 h post transfection. Cells were lysed in phosphatase assay buffer (50 mM Hepes, pH 7.5, 100 mM NaCl, 2 mM MgCl_2_, 1 mM dithiothreitol (DTT) and 0.5% NP-40) supplemented with protease inhibitors and phosphatase inhibitors (P0044 and P5726 from Sigma) to preserve phosphorylation. Lysate was incubated with Anti-Flag M2 affinity gel (A2220 from Sigma) at 4 °C with rotation overnight. Following 4–5 washes, Flag-S–ULK1 substrate was eluted in phosphatase assay buffer containing 400 μg ml^−1^ 3 × FLAG peptide (Sigma).

The Flag-S–ULK1 substrate was stable in reactions containing cell lysate but not in buffer alone when incubated at 30 °C. To stabilize the substrate in reactions that use recombinant phosphatases, we used ULK1/2 DKO MEF cell lysate devoid of phosphatase activity. To generate this protective lysate, we incubated ULK1/2 DKO MEF cell lysate (in 20 mM Hepes pH 7.5, 50 mM KCl, 5 mM Pi, 1 mM DTT + 0.5% NP-40 buffer) with hydroxyapatite beads at 4 °C overnight with rotation. Approximately 50 μl bead volume was used per mg of total cell lysate. Protective lysate was tested for ability to stabilize Flag-S–ULK1 substrate and lack of remnant phosphatase activity before use in assays (Fig. 4a). Incubation with hydroxyapatite beads was repeated if phosphatase activity was not completely depleted. Protective lysate was concentrated in a Amicon Ultra-4 centrifugal filter unit (UFC801024 from EMD Millipore) if total protein concentration was too low.

For phosphatase assays with cell lysate: Flag-S–ULK1 substrate was incubated with 10 μg of total cell lysate at 30 °C for the indicated amount of time. For phosphatase assays with recombinant proteins or for biochemical purification of B55α subunit: Flag-S–ULK1 substrate plus 7–10 μg of phosphatase-depleted protective lysate was incubated with recombinant proteins or column eluted fractions at 30 °C for 20 min.

### Biochemical purification of B55α subunit

A 20 ml cell pellet of starved ULK1/2 DKO MEF cells was lysed by douncing in 90 ml of Buffer A (20 mM Hepes pH 7.5, 10 mM NaCl and 1 mM DTT). Cell lysate was centrifuged at 12,000*g* for 10 min at 4 °C. Supernatant was further clarified by centrifugation at 100,000*g* for 1 h at 4 °C. Clarified cell lysate was loaded into SP column. Flow-through from SP column was collected and loaded into Heparin column. Flow-through from Heparin column was collected and loaded into Q-column (Buffer A: 20 mM Hepes pH 7.5, 10 mM NaCl, 1 mM DTT; Buffer B: 20 mM Hepes pH 7.5, 1,000 mM NaCl, 1 mM DTT). Q-column was washed with 15% B and eluted with 35% Buffer B. Ammonium sulfate was added to a final concentration of 20% and incubated at 4 °C for 30 min. Precipitated proteins were removed by centrifugation at 47,800*g* for 10 min. Supernatant was collected and diluted to 10% ammonium sulfate before loading into a phenyl sepharose column (Buffer A: 5 mM Hepes pH 7.5, 1 mM DTT; Buffer B: 20% ammonium sulfate, 20 mM Hepes pH 7.5, 400 mM NaCl, 1 mM DTT). Phenyl sepharose column was eluted with a gradient of 50 to 0% Buffer B across 20 fractions. Fractions were dialysed and assayed for activity. Active fractions were combined and loaded into hydroxyapatite column (Buffer A: 20 mM Hepes pH 7.5, 10 mM NaCl, 1 mM DTT; Buffer B: 1 M Pi, 1 mM DTT pH 7.5). Column was washed with 3% Buffer B and eluted with a gradient of 3 to 18% Buffer B across 20 fractions. Fractions were dialysed and assayed for activity. Active fractions were combined and concentrated with a Q-column. Gel filtration column was run and active fractions were combined and loaded into a mono-Q-column (same buffer conditions as Q-column). Mono-Q-column was washed with 18% Buffer B (180 mM NaCl) and eluted in 20 × 1 ml fractions across a 19% Buffer B (190 mM NaCl) to 34% Buffer B (340 mM NaCl) gradient. Eight microlitre of each fraction was assayed for activity.

### Purification of recombinant proteins

The full length of human PP2A catalytic subunit (PP2AC, α isoform NM_002715) or regulatory subunit B55α (human, NM_002717) was cloned into baculovirus transfer vector pFastBac as N-terminal 9 × His-tagged proteins. Full-length human PP2A scaffolding subunit (PRL65, α isoform NM_014225) or mouse Alpha4 (NM_008784) were cloned into pFastBac as N-terminal 9 × His and Flag tandem-tagged proteins. Recombinant baculovirus was produced using Bac-to-Bac expression system from Invitrogen. PP2AC and PRL65 were co-expressed in a suspension culture of Sf9 cells for co-purification. Briefly, cell pellets were lysed by douncing and purified by Ni-NTA resin, followed by gel filtration to isolate fractions containing both PP2AC and PRL65. PP2AC and Alpha4 were co-expressed in a suspension culture of Sf9 cells for co-purification. Cell pellets were lysed by douncing and purified by Ni-NTA resin, followed by incubation with Anti-Flag M2 affinity gel (A2220 from Sigma) at 4 °C for 5 h. PP2AC bound to Alpha4 was eluted with 3 × Flag peptide and assayed for activity immediately. B55α was expressed in Sf9 cells and purified by Ni-NTA resin followed by ion exchange chromatography through a Q-column.

### Knockdown by shRNA or siRNA

The following shRNA constructs were purchased from Sigma: pLKO non-target control (CAACAAGATGAAGAGCACCAA), pLKO mPP2AC shRNA (GCGACATTGTTGGTCAAG AAT), pLKO mPRL65 shRNA (GCCACCAGCAACCTTAAGAAA), pLKO mPP1C shRNA (CCAGATCGTTTGTACAGAAAT), pLKO hULK1 shRNA (GCCCTTTGCGTTATATTGT AT). The following shRNAs were cloned into pLKOtet-ON inducible shRNA plasmid system purchased from Addgene: mPP2AC shRNA (GCGACATTGTTGGTCAAGAAT), hB55α sh 1 (TCCTGCTTAGTTGAGATAGTT), hB55α sh 2 (GTAGATGATGATGTAGCAGAA). Lenti- viruses were made by co-transfecting shRNA constructs and packaging vectors into 293Ts. Lentiviral preparations were passed through a 0.45 μM Polyethersulfone membrane filter (PES) filter and supplemented with polybrene before being used to transduce cells. Stable cell lines were typically assayed for knockdown 48–72 h post transduction. For inducible knockdown, cell lines were grown in complete media containing 100 ng ml^−1^ doxycycline 48–72 h before assaying for knockdown.

For knockdown by siRNA, siGENOME Control siRNA (D-001210-05-20) and siGENOME SMARTpool FIP200 (RB1CC1) siRNA (M-021117-01) were purchased from Dharmacon. siRNAs were transfected into cells at a final concentration of 100 nM with 2 μl Lipofectamine 2,000 (Invitrogen) as per manufecturer’s protocol. Cells were assayed 48 h after transfection.

### Expression constructs

Autophagy marker LC3 was subcloned into pBabe/blast vector containing N-terminal GFP tag. pMIC vector was made by modifying pMIG vector (Addgene) to replace GFP in the backbone with mCherry. The coding sequence of human B55α, human PP2AC, and mouse Alpha4 were cloned into pMIC vector along with an N-terminal Flag-S tandem tag. pMSCV GFP-B55α and pMSCV GFP-PR72 used in immunoprecipitation experiments were a generous gift from Dr Emily Foley, Memorial Sloan-Kettering Cancer Center, New York, USA.

### Immunoprecipitations

Constructs expressing Flag-S–ULK1 and GFP-B55α or GFP-PR72 were co-transfected into 293Ts using Lipofectamine 2,000 (Invitrogen) according to the manufacturer’s recommendations. 24–48 h post transfection, cells were washed twice in cold PBS(−) and lysed in immunoprecipitation (IP) buffer (20 mM Tris pH 7.5, 150 mM NaCl, 1 mM DTT and 0.5% Triton X-100) supplemented with protease inhibitors. Cell lysate was centrifuged at 20,000*g* for 10 min at 4 °C and incubated with 10 μl of S-protein agarose beads (69704 from EMD Millipore) in a total volume of 1 ml overnight at 4 °C with rotation.

For pull down of Flag-S–PP2A complexes: Following treatment, Flag-S–PP2AC expressing cells were washed twice in cold PBS(−) and lysed in IP buffer supplemented with protease inhibitors. Cell lysate was centrifuged at 20,000*g* for 10 min at 4 °C. Protein concentration of clarified cell lysate was determined by Bio-Rad Protein Assay Dye reagent. An equal amount of clarified cell lysate from each treatment condition was incubated with 10 μl of S-protein agarose beads (69704 from EMD Millipore) for 4 h at 4 °C with rotation. For IP with MCLR beads (16–147 from EMD Millipore), cells were washed twice in cold PBS(−) and lysed in IP buffer supplemented with protease inhibitors. Cell lysate was centrifuged at 20,000*g* for 10 min at 4 °C. 500 μg of clarified cell lysate was incubated with 10 μl of MCLR beads for 4 h at 4 °C with rotation. In all cases, beads were then washed 5 times in IP buffer before elution in SDS-loading dye and analysis by SDS–PAGE.

For pull down of endogenous PP2A complexes: following treatment, cells were washed twice in cold PBS(−) and lysed in KCl–IP buffer (20 mM Tris pH 7.5, 100 mM KCl, 0.5 mM DTT, 0.2% NP-40 and 10% glycerol) supplemented with protease inhibitors. Cell lysate was centrifuged at 20,000*g* for 10 min at 4 °C. Protein concentration was measured. An equal amount of clarified cell lysate from each treatment condition (500 μg–1 mg) was incubated with 0.6–1 μl of Mouse anti-PP2AC antibody (clone 1D6 #05-421 from Millipore) at 4 °C with rotation overnight. Lysates were then incubated with Protein G agarose for 3 h at 4 °C with rotation. For 293Ts overexpressing Alpha4, PP2A IP was carried out 48 h post transfection.

For DSP (Lomant’s reagent, Thermo Scientific #22585) crosslinking in 293T cells: cells were grown in 10-cm dishes, treated and lysed in 1 ml DSP–IP buffer (20 mM Hepes pH 7.5, 100 mM KCl, 1 mM Mn^2+^ and 0.1% NP-40) supplemented with protease inhibitors. Cell lysate was centrifuged at 20,000*g* for 10 min at 4 °C. One millilitre of clarified cell lysate was gently added to 4 μl of crosslinking reagent DSP (125 mg ml^−1^ stock in DMSO) to make a final concentration of 0.5 mg ml^−1^ DSP. This was rotated at room temperature for 4 min. Reaction was quenched by the addition of 25 μl of 1 M Tris pH 7.5. Lysates were then centrifuged at 20,000*g* for 10 min at 4 °C to remove any precipitated DSP. Protein concentration of clarified lysates was measured. One-milligram crosslinked cell lysate was incubated with 1 μl anti-PP2AC antibody (clone 1D6) and immunoprecipitation was carried out as described above. Crosslinking was reversed prior to gel run by boiling samples 3 × 10-min in SDS-loading dye supplemented with additional β-mercaptoethanol (25% V/V). After gel run, PAGE gel was rinsed in transfer buffer for 15–30 min before transfer.

### Cell growth assay

Cells were plated on a 12-well plate in triplicate at 15,000 cells per well. The day after plating was considered day 0. Cells were fixed in 10% formalin for 10 min and stained with 0.1% crystal violet for 20 min. Excess dye was removed by washing three times with water. Stained cells were left to air dry before dye was extracted with 10% acetic acid. Relative proliferation was determined by measuring absorbance at 570 nm.

### Soft agar assay

Soft agar assay was carried out in 6-well plates in triplicate for each cell line. An underlay of 2 ml media with 0.5% agar (2-Hydroxyethylagarose, Sigma A4018) was dispensed into each well and allowed to set. Cells were resuspended in media containing 0.4% agar and seeded at 5,000 cells per well in a volume of 1.5 ml. After 10–14 days, colonies were stained with iodonitrotetrazolium chloride (Sigma, I10406) and visualized with GELCOUNT imaging system (Oxford Optronix). Total colony number for each well was enumerated based on colony size and staining intensity.

## Additional information

**How to cite this article:** Wong, P.M. *et al.* Regulation of autophagy by coordinated action of mTORC1 and protein phosphatase 2A. *Nat. Commun.* 6:8048 doi: 10.1038/ncomms9048 (2015).

## Supplementary Material

Supplementary Information Supplementary Figures 1-8
