# Supplementary material for: Regulation of autophagy by coordinated action of mTORC1 and protein phosphatase 2A
Source: Nat Commun. 2015 Aug 27;6:8048. doi: 10.1038/ncomms9048 (PMC4552084; doi:10.1038/ncomms9048)
Supplement: Supplementary Information — Supplementary Figures 1-8 [file ncomms9048-s1.pdf]

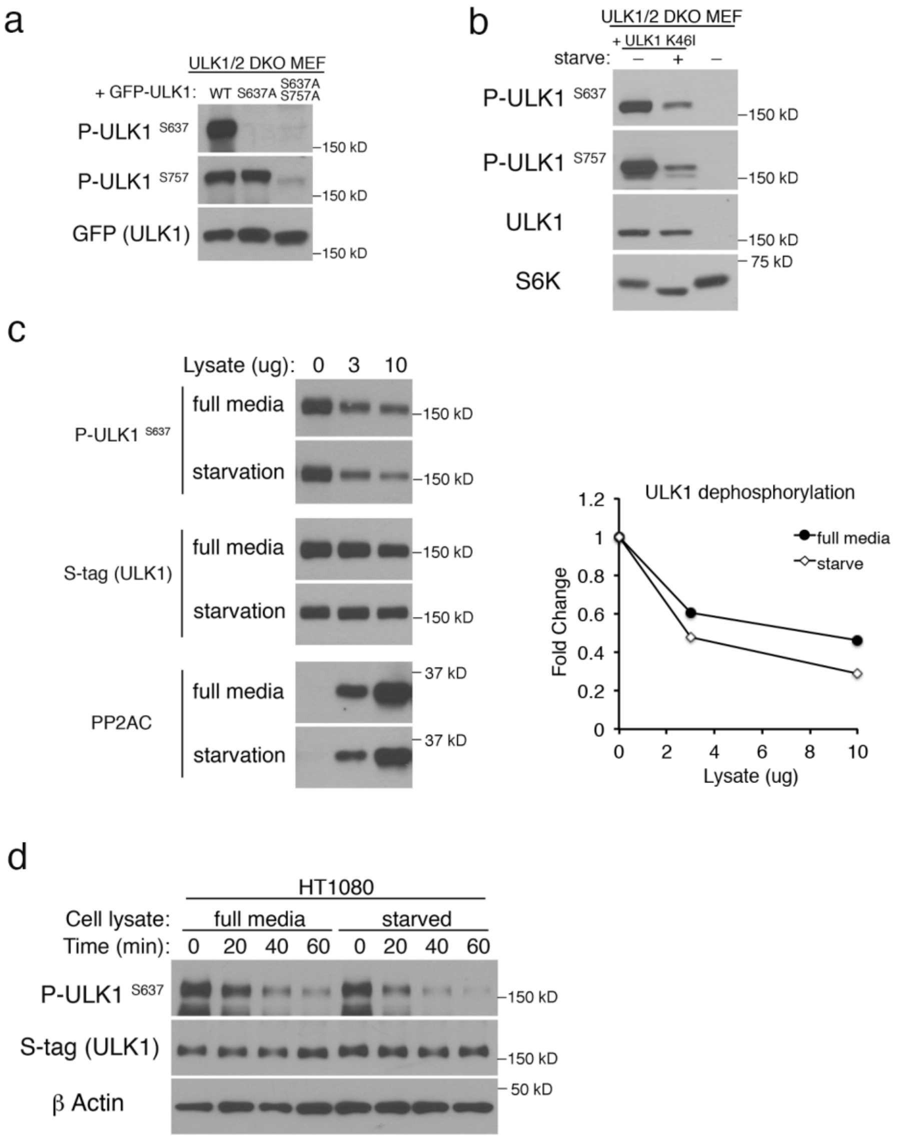

Supplementary Figure 1 Phosphatase activity is required for starvation induced autophagy. (**a, b**) ULK1 S637 and S757 phospho antibodies are specific. In (a), the antibodies cannot recognize ULK1 when the respective sites of ULK1 (S637, S757) are mutated to alanine. In (b), the antibodies recognized ectopic ULK1 (K46I mutant) expressed in ULK1/2DKO MEFs, and the signal was reduced upon starvation, correspondent with dephosphorylation of ULK1. (**c**) Starvation increases phosphatase activity in MEF cells. In vitro phosphatase assay was carried out using Flag-S-ULK1 as a substrate and increasing amounts of total cell lysate from MEFs that were kept in full media or starvation media. Reactions were incubated at 30°C for 30 min. (**d**) Starvation increases phosphatase activity in HT1080 cells. In vitro phosphatase assay was carried out using 10 μg of total cell lysate from HT1080 cells that were kept in full media or starvation media. Reactions were terminated at the indicated time.

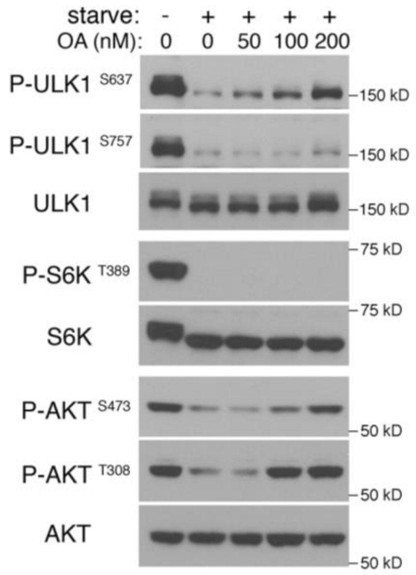

Supplementary Figure 2 Okadaic acid (OA) inhibits the dephosphorylation of ULK1 at S637 in a dose dependent manner. MEF cells were incubated in starvation media with increasing amounts of OA for 1 hr. Phosphorylation on ULK1 and S6K were monitored using site specific phospho-antibodies. AKT, previously reported as OA sensitive, was included as a positive control for OA treatment.

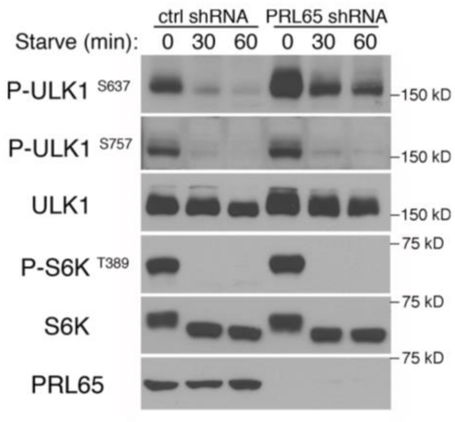

Supplementary Figure 3 PRL65 is required for ULK1 S637 dephosphorylation. MEF cells were transduced with lentivirus containing control shRNA or shRNA targeting PRL65, the scaffolding subunit of PP2A. 48-72 hrs post transduction, cells were incubated in starvation media for the indicted time and analysed by immunoblotting.

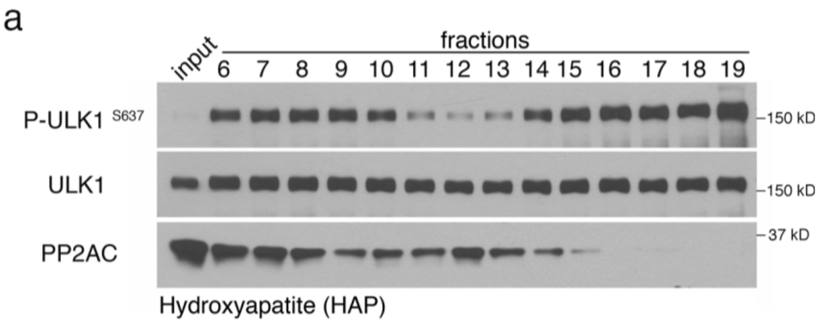

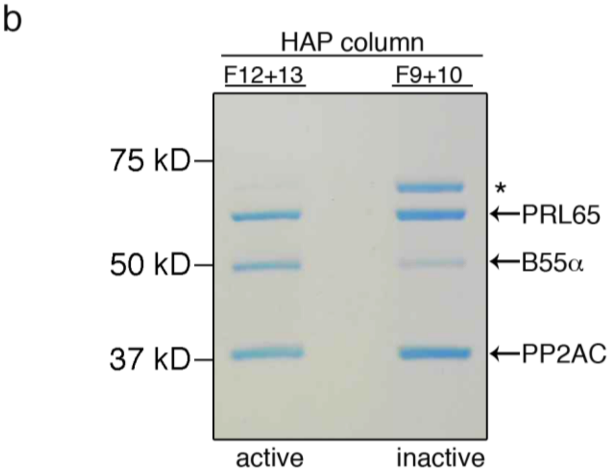

c

Serine/threonine-protein phosphatase 2A 56 kDa regulatory subunit gamma isoform (PPP2R5C)

1 MLTCNKAGSG MVVDAASSNG PFQPVALLHI RDVPPADQEK LFIQKLR**QCC**
51 **VLFDFVSDPL SDLK**WKEVKR AALSEMVEYI THNRNVITEP IYPEAVHMFA
101 VNMFR**TLPPS SNPTGAEFDP EED**EPTLEAA WPHLQLVYEF **FLRFLESPDF**
151 **QPNIAKK**YID QKFVLQLLEL FDSEDPRERD FLKTTLHR**IY GKFLGLR**AYI
201 RKQINNIFYR FIYETEHHNG IAELLEILGS IINGFALPLK EEHKIFLLK**V**
251 **LLPLHKVKSL SVYHPQLAYC VVQFLEK**DST LTEPVVMALL KYWPKTHSPK
301 EVMFLNELEE ILDVIEPSEF VKIMEPLFRQ LAK**CVSSPHF QVAER**ALYYW
351 NNEYIMSLIS DNAAK**ILPIM FPSLYR**NSKT HWNK**TIHGLI YNALKLFMEM**
401 **NQK**LFDDCTQ QFKAEKLKEK LKMKEREEAW VKIENLAKAN PQAQKELKKD
451 RPLVRRKSEL PQDPHTEKAL EAHCRASELL SQDGR

sequence coverage: 24%

Supplementary Figure 4 Only a subset of PP2A complexes in the cell can dephosphorylate ULK1. (**a**) Not all fractions containing PP2AC have activity against ULK1. Fractions from Hydroxyapatite column were assessed for activity against ULK1 and presence of PP2AC. Active fractions (11-13) were combined as input for the next purification step. (**b, c**) Regulatory subunit purified from inactive fraction of HAP column. (b) shows a Coomassie blue gel of PP2A purified from active and inactive fractions of the HAP column. * indicates unknown band that was excised and analyzed by mass spectrometry, which was identified as the B'γ regulatory subunit. (c) shows results of mass spectrometry analysis. Peptide fragments matched to the B'γ regulatory subunit of PP2A with a sequence coverage of 24%. Identified proteins: At p<0.01, average false discovery rate, FDR: 0%.

a

Serine/threonine-protein phosphatase 2A catalytic subunit beta isoform (PPP2CB)

1 **MDDKAFTK**EL DQWVEQLNEC KQLNENQVRT LCEKAKEILT KESNVQEVRC
51 PVTVCGDVHG QFHDLMELFR IGGK**SPDTNY LFMGDYVDR**G YYSVETVTLL
101 VALKVRYPER ITILRGNHES RQITQVYGFY DECLR**KYGNA NVWK**YFTDLF
151 DYLPLTALVD GQIFCLHGGL SPSIDTLDHI RALDR**LQEVP HEGPMCDLLW**
201 **SDPDDRGGWG ISPRGAGYTF GQDISETFNH ANGLTLVSR**A HQLVMEGYNW
251 CHDRNVVTIF SAPNYCYR**CG NQAAIMELDD TLKYSFLQFD PAPR**RGEPHV
301 TR**RTPDYFL**

sequence coverage: 38.5%

b

Serine/threonine-protein phosphatase 2A 65 kDa regulatory subunit A alpha isoform (PPP2R1A)

1 MAAADGDDSL YPIAVLIDEL RNEDVQLRLN SIK**KLSTIAL ALGVER**TRSE
51 LLPFLTDTIY DEDEVLLALA EQLGTFTTLV GGPEYVHCLL PPLESLATVE
101 ETVVRDKAVE SLR**AISHEHS PSDLEAHFVP LVKRLAGGDW FTSR**TSACGL
151 FSVCYPR**VSS AVKAELR**QYF R**NLCSDDTPM VRR**AAASKLG EFAK**VLELDN**
201 **VKSEIIPMFS NLASDEQDSV R**LLAVEACVN IAQLLPQEDL EALVMPTLRQ
251 AAEDKSWR**VR YMVADKFTEL QKAVGPEITK TDLVPAFQNL MKDCEAEVR**A
301 AASHKVKEFC ENLSADCREN VIMTQILPCI K**ELVSDANQH VK**SALASVIM
351 GLSPILGKDN TIEHLLPLFL AQLKDECPEV RLNIISNLDC VNEVIGIRQL
401 SQSLLPAIVE LAEDAKWRVR LAIIEYMPLL AGQLGVEFFD EKLNSLCMAW
451 LVDHVYAIRE AATSNLKKLV EK**FGKEWAHA TIIPKVLAMS GDPNYLHR**MT
501 TLFCINVLSE VCGQDITTK**H MLPTVLRMAG DPVANVR**FNV AKSLQK**IGPI**
551 **LDNSTLQSEV KPILEKLTQD QDVDVK**YFAQ EALTVLSLA

sequence coverage: 37.2%

c

Serine/threonine-protein phosphatase 2A 55 kDa regulatory subunit B alpha isoform (PPP2R2A)

1 M**AGAGGGNDI QWCFSQVK**GA VDDDVAEADI ISTVEFNHSG ELLATGDKGG
51 R**VVIFQQEQE NKIQSHSRGE YNVYSTFQSH EPEFDYLKSL EIEEKINK**IR
101 WLPQK**NAAQF LLSTNDKTIK** LWKISER**DKR PEGYNLKEED GRYRDPTTVT**
151 **TLRVPVFRPM DLMVEASPR**R IFANAHTYHI NSISINSDYE TYLSADDLR**I**
201 **NLWHLEITDR** SFNIVDIKPA NMEELTEVIT AAEFHPNSCN TFVYSSSK**GT**
251 **IRLCDMR**ASA LCDR**HSKLFE EPEDPSNR**SF FSEIISSISD VKFSHSGRYM
301 MTRDYLSVKI WDLNMENRPV ETYQVHEYLR SKLCSLYEND CIFDKFECCW
351 NGSDSVVMTG SYNNFFRMFD RNTK**RDITLE ASRENNKPR**T VLKPRKVCAS
401 GKRK**KDEISV DSLDFNKKIL HTAWHPK**ENI IAVATTNNLY IFQDKVN

sequence coverage: 43.2%

Supplementary Figure 5 Mass spectrometry analysis of p36, p65 and p50 bands from ULK1 phosphatase purification. The peptides detected during mass spectrometry analysis are shown in red. (**a**) The p36 band produced peptide fragments that matched to the catalytic subunit of PP2A with a sequence coverage of 38.5%. (**b**) Peptide fragments from the p65 band matched to the scaffolding subunit of PP2A with a sequence coverage of 37.2%. (**c**) Peptide fragments from the p50 band matched to the B55α regulatory subunit of PP2A with a sequence coverage of 43.2%. Identified proteins: At $p<0.01$, average false discovery rate, FDR: 0%.

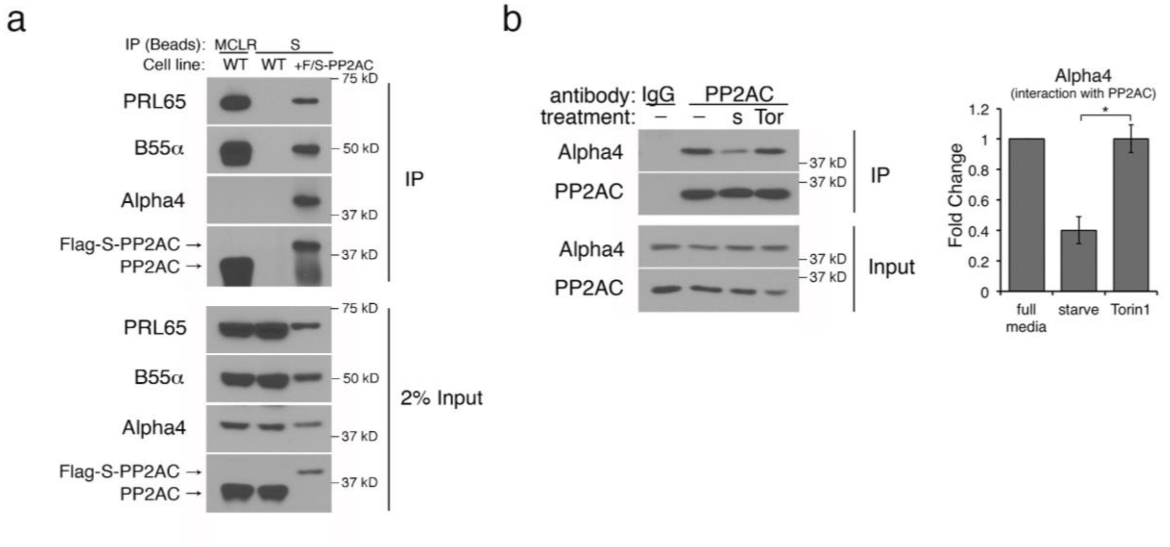

Supplementary Figure 6 Starvation induces Alpha4 dissociation from PP2AC. (**a**) Endogenous PP2AC-Alpha4 complex is catalytically inactive. WT MEFs (ctrl) were lysed and incubated with microcystin-LR (MCLR) beads which bind to the catalytic pocket of PP2AC. As a comparison, MEFs expressing Flag-S-PP2AC were lysed and incubated with S-beads to pull down total PP2A complexes in the cell. (**b**) WT MEFs were incubated in starvation media or media containing 1 μM Torin1 for 1 hour, lysed and incubated with control antibody or antibody against PP2AC. The amount of Alpha4 interacting with PP2AC under each condition was monitored by Immunoblotting and quantitated on the right (fold change relative to full media ± s.d., n=5. two-tail student's t-Test, * $p<0.05$).

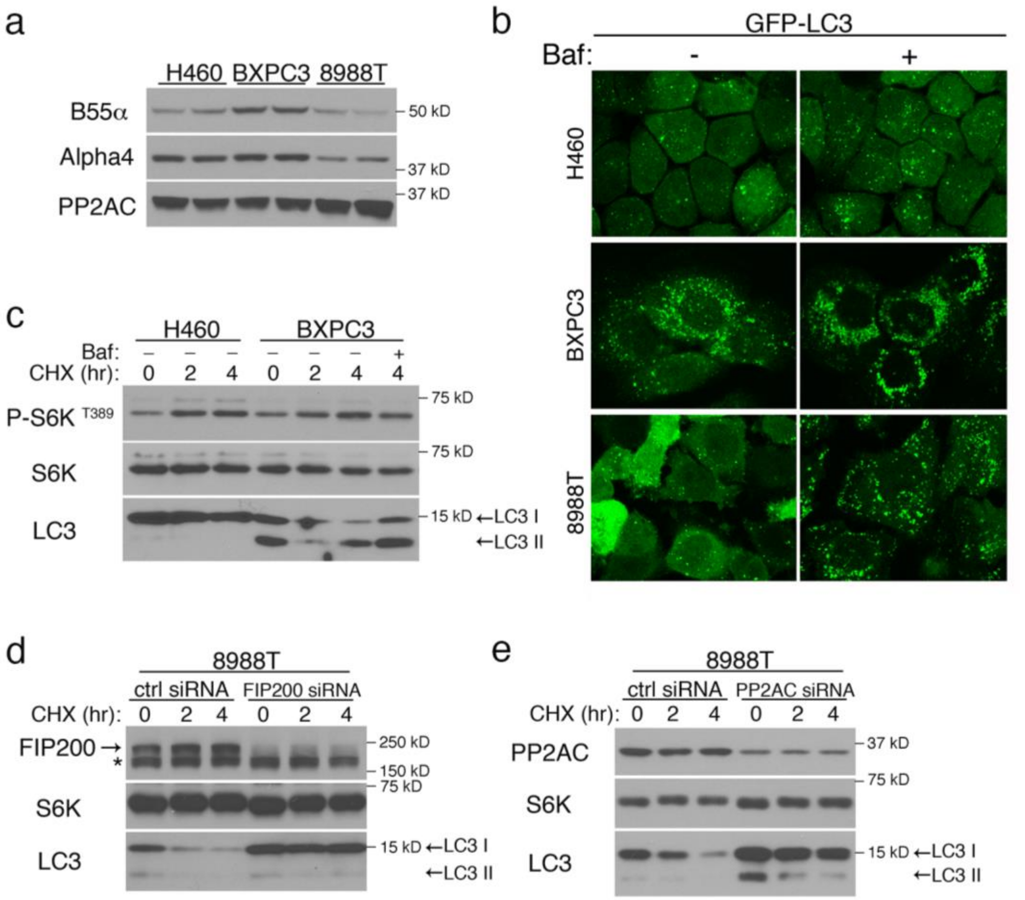

Supplementary Figure 7 High basal autophagy in Pancreatic Ductal Adenocarcinoma cell lines is ULK1 complex dependent. (**a**) Immunoblot for endogenous levels of PP2A regulatory proteins B55α and Alpha4 in control cell line H460 and PDAC cell lines BXPC3 and 8988T. (**b**) Cell lines stably expressing GFP-LC3 were kept in complete media in the presence or absence of 20 nM bafilomycin (Baf) for 90 min. Representative images from two independent experiments shown. (**c**) BXPC3 has high basal autophagy. BXPC3 and H460 were kept in complete media with 20 μg ml^{-1} cycloheximide (CHX) for the indicated amount of time. Where indicated, 10 nM bafilomycin was added at the start of CHX treatment. Cells were lysed and immunoblotted for endogenous LC3. (**d**) FIP200 is required for basal autophagy in 8988T cells. 8988T was transduced with control siRNA or siRNA targeting FIP200, another member of the ULK1 complex. Cells were then treated as in (c). * indicates non-specific band. (**e**) PP2AC is required for basal autophagy in 8988T cells. 8988T was transduced with control siRNA or siRNA targeting the catalytic subunit of PP2A. Cells were then treated as in (c).

Figure 1c

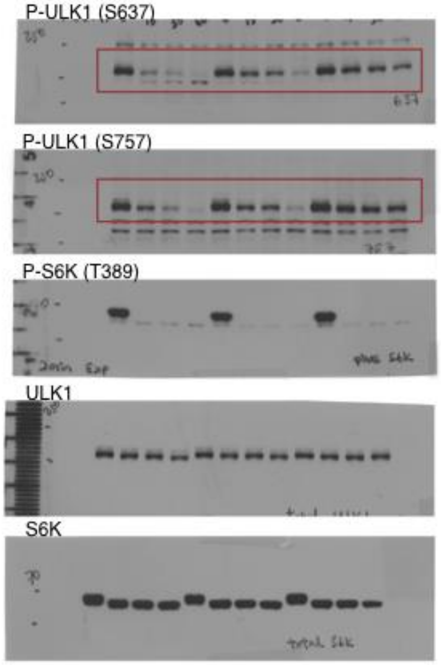

Figure 1e

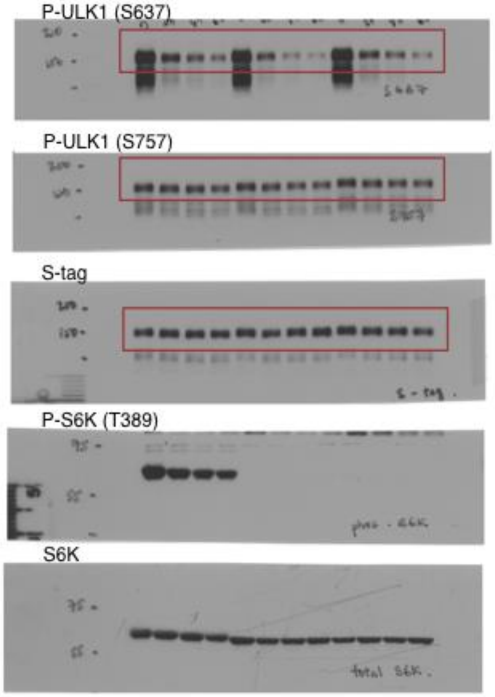

Figure 2a

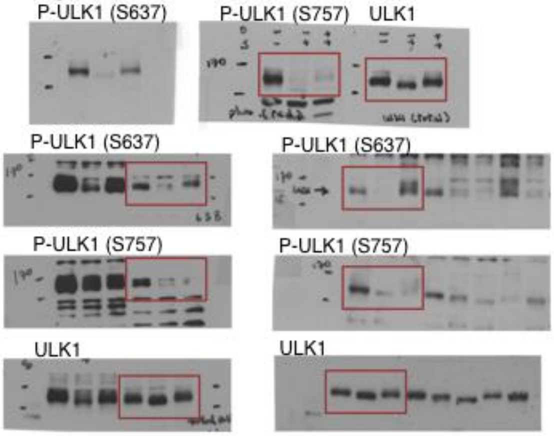

Figure 2b

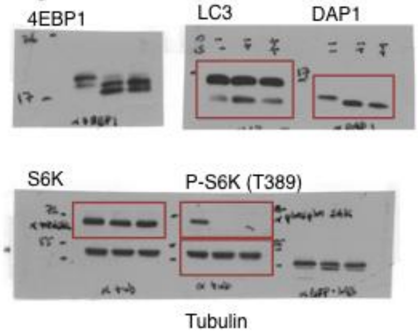

Figure 3a

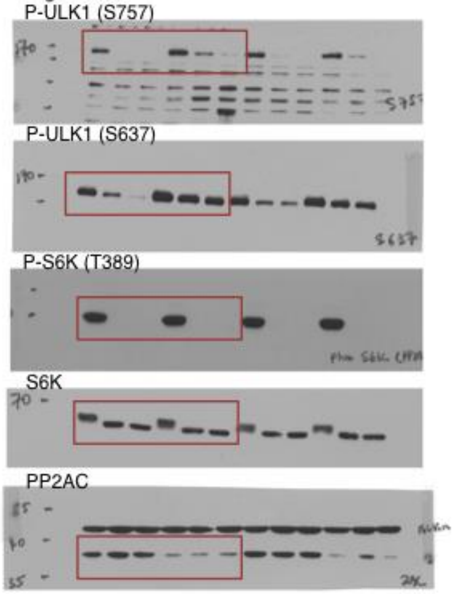

Figure 3c

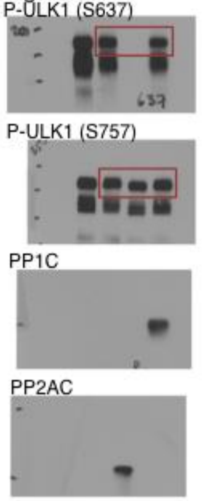

Figure 5d

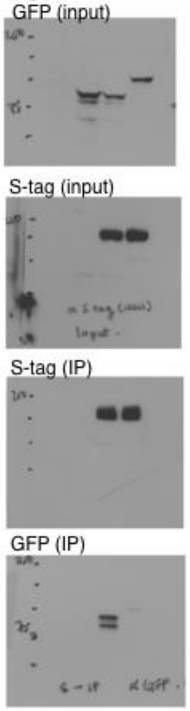

Figure 4e

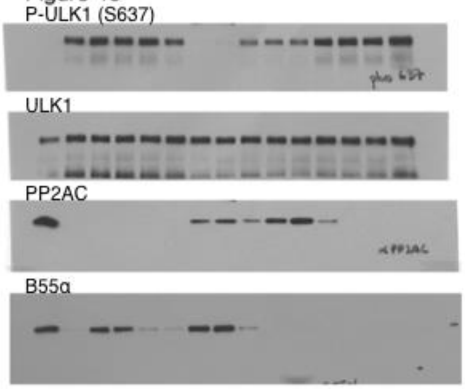

Figure 5b

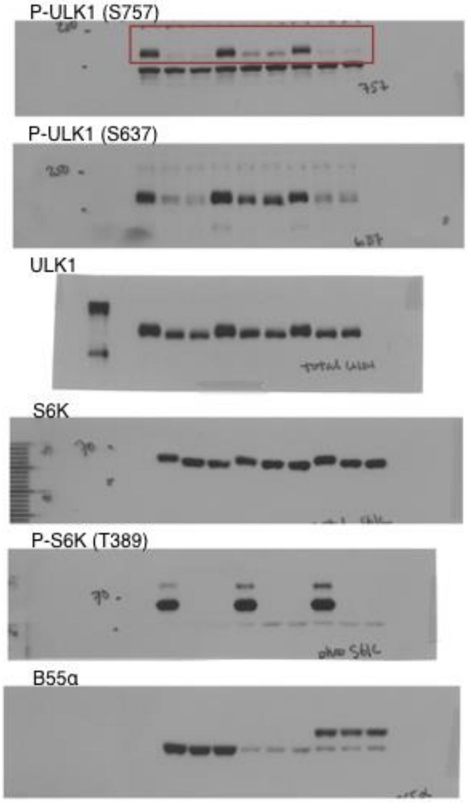

Figure 6B

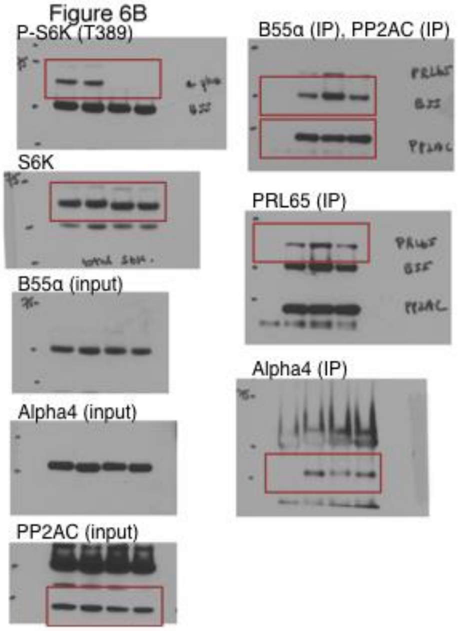

Figure 6C

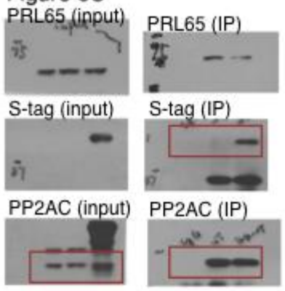

Figure 6D

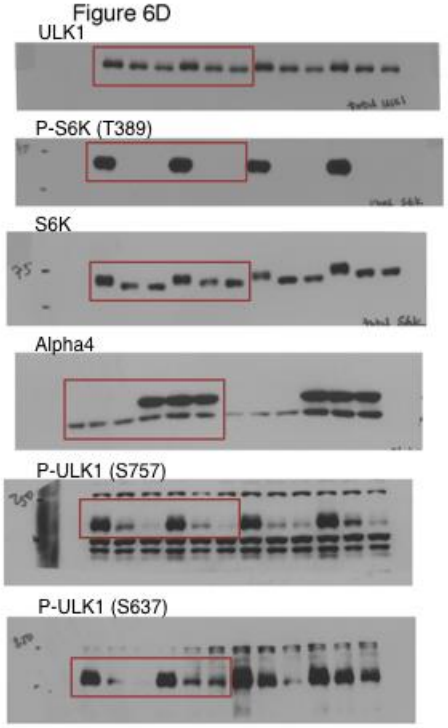

Figure 7a

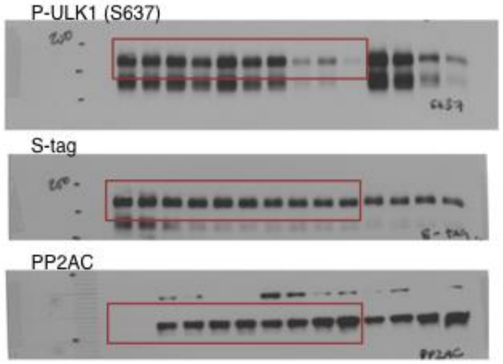

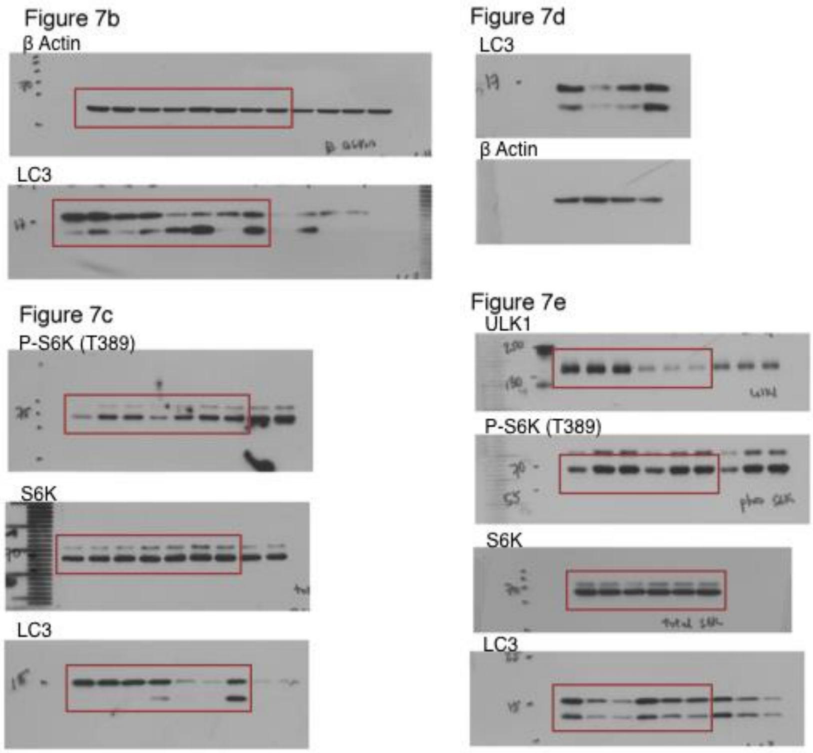

Supplementary Figure 8 Full scans of western blots related to respective figures as indicated. The protein of interest being detected is labeled on the top left hand corner of each blot.
